# Adequacy of iodized salt and its associated factors among households in the Bahir Dar Zuria district, Northwest Ethiopia, 2022

**DOI:** 10.3389/fnut.2023.1215613

**Published:** 2023-10-27

**Authors:** Emebet Chalachew Temech, Oumer Said, Genete Endalik, Yeshalem Mulugeta Demilew, Mahider Awoke Belay, Tadele Derbew Kassie, Anteneh Mengist Dessie

**Affiliations:** ^1^Bahir Dar Zuria District Health Office, Bahir Dar, Ethiopia; ^2^Department of Nutrition and Dietetics, School of Public Health, College of Medicine and Health Science, Bahir Dar University, Bahir Dar, Ethiopia; ^3^Department of Public Health, College of Medicine and Health Science, Injibara University, Injibara, Ethiopia; ^4^Department of Public Health, College of Health Science, Debre Markos University, Debre Markos, Ethiopia; ^5^Department of Public Health, College of Health Science, Debre Tabor University, Debre Tabor, Ethiopia

**Keywords:** adequacy, availability, iodized salt, households, Bahir Dar Zuria, Ethiopia

## Abstract

**Background:**

The inadequacy of iodine in salt is the a contributing factor behind lack of awareness and poor economic performance in developing countries. To address the issue of iodine deficiency, universal salt iodization has been implemented globally. Nevertheless, it is imperative to closely monitor the sufficiency of iodine in salts to achieve its intended objective at the household level in the Bahir Dar Zuria district.

**Objective:**

To assess the adequacy of iodized salt and its associated factors among households in the Bahir Dar Zuria district, Northwest Ethiopia.

**Methods:**

A community-based cross-sectional study was conducted from May to June 2022 among households in Bahir Dar Zuria district. The data were gathered from 825 households that were chosen using a multistage sampling process. Iodometric titration was used to assess the amount of iodine in salt. The data were gathered using a structured questionnaire that was administered by an interviewer. For data entry and analysis, EpiData version 3.01 and SPSS version 25.0 were used, respectively. To evaluate the association between explanatory factors and the outcome variable, binary logistic regression was conducted, and significance was determined at alpha 0.05.

**Result:**

This study analyzed a total of 825 households. Of these, only 384 [46.5, 95% CI, 43.5–50.5%] households had adequately iodized salt at home. Age increase of 1 year [AOR = 1.04, 95% CI = 1.02–1.06], being an urban resident [AOR = 3.18, 95% CI = 1.84–5.48], diploma and above educational attainment [AOR = 3.74, 95% CI = 1.99–7.02], checking salt by asking the seller [AOR = 2.21, 95% CI = 1.26–3.88], storing salts in closed containers [AOR = 1.57, 95% CI = 1.13–2.19], and storing salts in a dry and cool area [AOR = 2.72, 95% CI =1.37–5.42] were associated with the adequacy of iodized salt at the household level.

**Conclusion and recommendation:**

The percentage of households in the district (46.5%) that had enough iodized salt in their homes is still extremely low and falls short of the targeted level for the country. At the household level, adequate iodized salt was found to be associated with age, place of residence, level of education, checking salt iodization while purchasing, place of salt storage, and cover use for salt containers. Therefore, increasing the accessibility of iodized salt at the household level is essential.

## Background

Iodine is a trace element for the synthesis of thyroid hormones, which are required for the body’s growth, development, and control of metabolic processes (1). Iodine Deficiency Diseases (IDDs) arise when the body does not receive the required quantity of iodine daily (2). IDD is a preventable public health problem on a global, regional, and national scale (3). More than 120 nations throughout the world use the well-established method of iodizing salt to avoid IDDs. At the household level, the iodine content of salt should be adequate [>15 parts per million (PPM)] (4). Ethiopia is a salt-producing country that has backed a mandated salt iodization effort and is working with UNICEF and Micronutrient initiatives to boost iodized salt use to >90%, lowering Iodine Deficiency (ID) rates (5).

Globally, more than 25% of the world’s population suffers from iodine deficiency illnesses, which remain a severe public health concern (3, 6). The number of iodine-deficient countries has decreased during the past decade from 54 to 25, while the number of countries with a sufficient intake of iodine increased from 67 to 116 (7). The percentage of people who consume enough iodized salt varies from 2 to 98% at the national and neighborhood levels (4). The combined estimate of goiter among Ethiopian children was 40.50%, and Ethiopian women had a cumulative goiter rate of more than 35.8% nationwide (8).

Inadequate iodine consumption or storage can lead to psychological growth abnormalities (cretinism, dwarfism), goiter, muscular dystrophy, spontaneous miscarriages, and hypothyroidism (2, 9). Low dietary iodine intake is the primary cause of iodine insufficiency. Populations are frequently impacted when the iodine content of the soil is reduced as a result of repeated water leaching and severe rainfall. Such soil does not contain enough iodine for crops to grow (10). The factors affecting inadequate intake of iodized salt in households are age, sex, occupational status, educational status, religion (11), salt container, knowledge regarding iodized salt availability (12), residence, duration of storage, and checking the status of salt while purchasing (13, 14).

In 2011 and 2015, the Ethiopian government demonstrated its unwavering commitment to battling ID by implementing mandatory universal salt iodization and a health sector transformation strategy to make iodized salt available to 80% of households. With the second National Nutrition Programme 2016–2020 and the 2019 National Food and Nutrition Policy playing a crucial role in tackling numerous nutrition concerns, Ethiopia has a rich nutrition policy environment (15). In 2016, the government established the second National Nutrition Program (NNP) cycle, which focused on the first 1,000 days of life, to control micronutrient deficiencies in the most vulnerable populations by 2030, one of the program’s main goals (16). However, the prevalence of IDD is more common in the research location, showing that iodized salt will not be available at the household level (17).

To the best of our knowledge, no research using the titration approach has been done in the current study area to evaluate how well-iodized salt is used in households. The Iodometric Titration Method (gold standard) (IDTM) (18) was used to measure the amount of iodine present in the salt used in each household; therefore, this study aims to determine the adequacy of iodized salt and its associated factors at the household level in the Bahir Dar Zuria district, Northwest Ethiopia, 2022.

## Materials and Methods

### Study design, area, and period

A community-based, cross-sectional study was conducted in the Bahir Dar Zuria district of Northwest Ethiopia from May 20 to June 30, 2022. The Bahir Dar Zuria district is one of the 14 districts in the West Gojjam Zone and is located around Bahir Dar City, approximately 560 kilometers from Addis Ababa. The district is situated at an altitude of 1,700–2,300 meters above sea level. Approximately 1,035 mm of rain falls on the district each year on average. The minimum and maximum temperatures are 10°C and 32°C, respectively. According to a survey of the land in this woreda, 21% of it is arable, 9% is pasture, 8% is forest or shrubland, 36% is submerged under water, and the remaining 26% is unusable land.

### Sample size determination and sampling technique

The sample size was determined using the following presumptions and the formula for a single population proportion, $n=\frac{\left(Za/2\right)2\;P\left(1-P\right)}{d2}$, 4% with margin error, 28.9% projected percentage of households with adequate iodized salt, 95% confidence level (19). The total sample size is 850 when the design effect of 1.5 and the 15% non-response rate are taken into account. A multistage sampling procedure was used to choose the study participants. From a total of 36 rural and urban kebeles, 5 rural and 4 urban kebeles were randomly chosen by lottery method. Based on the size of the households, the sample size was proportionally assigned to each of the chosen kebeles, and households from each of the kebeles were again chosen using systematic random sampling techniques.

### Data collection tools and procedures

The data were gathered using a structured face-to-face interviewer-administered questionnaire that was tailored to the local context and adapted from pertinent research to incorporate all the factors in the evaluation tool. The data were gathered by nine experienced diploma nurses and five lab technicians, under the supervision of three BSc nurses. After carefully interviewing the respondents of the chosen households in the community, a 50 g sample of salt was carefully taken and placed in an airtight plastic bag. By releasing iodine from the salt and titrating the iodine with sodium thiosulphate while utilizing starch as an external indicator, the iodine content of the salt was discovered. The iodine levels of the salt samples were examined using an iodometric titration technique in the laboratory of the Ethiopian Public Health Institute. Approximately 3 tablespoons (50 g) of salt will dissolve in roughly one hundred milliliters (100 ml) of purified water. To convert all of the iodate present to free iodine, 30 mg of potassium iodide powder was added after the pH was brought down to 2.8 using 0.6% hydrochloric acid. Utilizing newly prepared starch as the endpoint indication, the freed iodine was titrated with a freshly prepared 0.004 M sodium thiosulphate solution. Iodine concentration in parts per million (PPM) was estimated using the standard conversion table for iodine determination, and the thiosulphate level in the burette was noted. Each household’s three triplicate samples had their mean calculated (20).

The wealth index was calculated using data on the possession of various consumer goods by the household, including television; number of oxen, sheep, and cars; and details about the home, including the type of flooring, the type of drinking water source, the number of restrooms, and other elements that were indicative of financial security. The assessment methods for urban and rural respondents are different and those tools were taken from the Ethiopian Demographic and Health Survey. The resulting asset scores were generated by the principal component analysis and were standardized to a normal distribution with a mean of zero and a standard deviation of one. These standardized scores are then used to establish the breakpoints that designate the three groups of wealth quintiles as poor, middle, and rich (16). The multicollinearity of the analysis was checked by the variance inflation factor (VIF).

### Operational definition

#### Iodine levels of the salt

If the tested salt had an iodine content ≥15 PPM, it was considered to be adequately iodized; nevertheless, if the result was <15 PPM, it was assumed to be inadequately iodized (21).

#### Knowledge about iodized salt

Participants were thought to have good knowledge of iodized salt if their scores for knowledge questions were above the mean and poor knowledge if their scores were below the mean (19).

### Data processing and analysis

After being coded, cleaned, and entered using EPI Data version 3.01, the data were transferred to SPSS version 25.0 for further processing and analysis. The frequency distribution, percentages, and proportions were ascertained using descriptive analysis. A binary logistic regression model was used to ascertain the associations between dependent and independent variables with a 95% confidence level. Adjusted odds ratio (AOR) with 95% CI in multivariable logistic regression and a *p*-value of <0.05 were used to determine the significant association, which included all explanatory factors with a *p*-value of <0.25 in the bivariable analysis. The odds ratio was used to determine how strongly the independent and dependent variables were associated. Hosmer and Lemeshow test (*p* = 0.11) was used to assess the model’s fitness.

## Results

### Socio-demographic characteristics of respondents

Out of 850 households, 825 respondents were interviewed, which gives a 97.1% response rate. Out of these participants, 748 (90.7%) were female respondents. The respondents’ average age was 33.15 + 8.84 years, with ages ranging from 19 to 63. The majority of respondents (95.6%) identified as Orthodox Christians; 85% were married; and 41.2% were illiterate. A total of 748 (78.5%) of the study’s households had families with less than five members (Table 1).

**Table 1:** Socio-demographic characteristics of respondents at households in Bahir Dar Zuria district, Northwest Ethiopia, 2022 (*n* = 825).

Variables	Number (%)
**Age**	
Less than 24	115 (13.9)
25–34	351 (42.6)
Greater than 34	359 (43.5)
**Sex**	
Male	77 (9.3)
Female	748 (90.7)
**Marital status**	
Married	701 (85)
Single	70 (8.5)
Divorced	17 (2.0)
Widowed	37 (4.5)
**Educational level**	
Unable to read and write	340 (41.2)
Able to read and write only	116 (14.1)
Elementary	133 (16.1)
High school and preparatory	136 (16.5)
College and above	100 (12.1)
**Occupation**	
Farmer	427 (51.8)
Housewife	169 (20.5)
Government employee	102 (12.3)
Others (self-employed and students)	127 (15.4)
**Place of residence**	
Rural	713 (86.4)
Urban	112 (13.6)
**Religion**	
Orthodox	789 (95.6)
Muslim	36 (4.4)
**Family size**	
<5 members	642 (77.8)
≥5 members	183 (22.2)
**Wealth index**	
Poor	157 (19.0)
Medium	114 (14.1)
Rich	552 (66.9)

### Knowledge about iodized salt and IDD

When asked if the respondents had ever heard of iodized salt, 825 (100%) responded that they had heard information about iodized salt from a variety of sources. Of them, 603 (73.1%) heard it from health professionals. A total of 486 (58.7%) respondents thought packed salt was iodized salt. However, 590 (71.5%) of respondents did not know that iodine deficiency disease is common in Ethiopia. According to this study’s findings, 442 respondents (or 53.6%) had overall good knowledge about iodized salt (Table 2).

**Table 2:** Knowledge of respondents regarding iodized salt and IDD in households in the Bahir Dar Zuria district, Northwest Ethiopia, 2022 (*n* = 825).

Variables	Number (%)
**Is packed salt iodized?**	
Yes	486 (58.9)
No	339 (41.1)
**Is non-packed salt iodized?**	
No	613 (74.3)
Yes	212 (25.7)
**Is iodine deficiency disease common in Ethiopia?**	
Yes	235 (28.5)
No	590 (71.5)
**Does iodine deficiency cause goiter?**	
Yes	484 (58.7)
No	341 (41.3)
**Does iodine deficiency cause mental retardation?**	
Yes	283 (34.3)
No	542 (65.7)
**Does iodine deficiency cause growth failure?**	
Yes	410 (49.7)
No	415 (50.3)
**Does iodine deficiency affect pregnant women?**	
Yes	498 (60.4)
No	327 (39.6)
**Can iodine deficiency be prevented by using iodized salt?**	
Yes	564 (68.4)
No	261 (31.6)
**Can iodine deficiency be prevented by eating seafood?**	
Yes	375 (45.5)
No	450 (54.5)
**Can iodine deficiency be prevented by eating eggs?**	
Yes	311 (37.7)
No	514 (62.3)
**Can iodine deficiency be prevented by eating dairy products?**	
Yes	410 (49.7)
No	415 (50.3)
**Can iodine deficiency be prevented by eating bread?**	
Yes	402 (48.7)
No	423 (51.3)
**Does iodized salt help keep you healthy?**	
Yes	569 (69.0)
No	256 (31.0)
**Is iodized salt important for human growth?**	
Yes	394 (47.8)
No	431 (52.2)
**Does iodized salt help to prevent iodine deficiency disease?**	
Yes	554 (67.2)
No	271 (32.8)
**Knowledge of iodized salt**	
Good knowledge	442 (53.6)
Poor knowledge	383 (46.4)

### Practice related to salt handling in the households

A total of 774 (93.8%) of the respondents said they kept their salt in a cool, dry location. However, 568 (68.8%) of the respondents kept the salt for more than 2 months after purchasing (Table 3).

**Table 3:** Salt handling practice of the respondents in households in the Bahir Dar Zuria district, Northwest Ethiopia, 2022 (*n* = 825).

Variables	Number (%)
**Where did you store the salt?**	
Dry and cool place	774 (93.8)
Moisture or fire area	51 (6.2)
**Did you expose the salt to sunlight?**	
No	712 (86.3)
Yes	113 (13.7)
**Did you wash the salt before use?**	
No	709 (85.9)
Yes	116 (14.1)
**Duration of salt storage in the household**	
Less than or equal to 2 months	257 (31.2)
Greater than 2 months	568 (68.8)
**Do you use a cover for the salt container?**	
Yes	576 (69.8)
No	249 (30.2)
**The practice of salt utilization**	
Good practice	482 (58.4)
Poor practice	343 (41.6)

### Accessibility and market related factors

A total of 433 (53%) respondents traveled less than 30 min to get iodized salt and 425 (51.5%) bought salt from the open market without reading the packs/labels (Table 4).

**Table 4:** Accessibility market related factors of iodized salt in households in the Bahir Dar Zuria district, Northwest Ethiopia, 2022 (*n* = 825).

Variable	Number (%)
**Is salt accessible in your area?**	
Yes	666 (80.7)
No	159 (19.3)
**Travel time to get iodized salt**	
<30 min	437 (53.0)
≥30 min	388 (47.0)
**How did you check iodized salt?**	
I did not check	79 (9.6)
Read the label on the pack	336 (40.7)
Asked the seller	410 (49.7)
**Where do you buy salt?**	
Open market	603 (73.1)
Near shop	222 (26.9)

### Factors associated with adequacy of iodized salt

Out of a total of 825 households, 384 (46.5%) [95% CI: 43.5–50.5%] have enough iodized salt at home, according to this study. Nine variables (age of the respondent, educational status, residence, knowledge about iodized salt and IDD, place of storage, duration of storage, cover use for salt container, place where they bought salt, and method of checking whether or not the salt is iodized) found to have a *p*-value of <0.25 in the bivariate binary logistic regression analysis were selected for the multivariable binary logistic regression model.

However, after adjusting for each of the previous variables using a multivariable binary logistic regression model, only the age of the respondent, educational status, residence, place of storage, cover use for the salt container, and method of checking whether the salt is iodized or not while buying were significant factors associated with the adequacy of iodized salt at a *p-*value < 0.05. Accordingly, the odds of having adequate iodized salt in the home increased by 4% for every year that the respondent’s age rose [AOR = 1.04, 95% CI = 1.02–1.06], and people who lived in urban areas were 3.18 times more likely to have adequate iodized salt than people who lived in rural areas [AOR = 3.18, 95% CI = 1.84–5.48]. The probabilities of having adequate iodized salt were 2.02 and 3.74 times higher in people with a primary education and a diploma or above, respectively [AOR = 2.02, 95% CI =1.26–3.24] and [AOR = 3.74, 95% CI = 1.99–7.02]. Similarly, participants who kept their salt in a dry, cool area and asked the seller to check the degree of iodization had probabilities of having adequate iodized salt that were 2.72 and 2.21 times higher, respectively, than those who did not [AOR = 2.72, 95% CI =1.37–5.42] and [AOR = 2.21, 95% CI = 1.26–3.88]. Compared to households that stored their salt in open containers, households that utilized covers on their salt containers were 57% more likely to have adequate iodized salt [AOR = 1.57, 95% CI = 1.13–2.19] (Table 5).

**Table 5:** Factor association with the adequacy of iodized salt in households in the Bahir Dar Zuria district, Northwest, Ethiopia, 2022 (*n* = 825).

Variables	Categories	Iodine titration result		COR (95% CI)	AOR (95% CI)
		Adequate	Inadequate		
Age in years	Treated as a continuous variable	33.15 ± 8.84		1.03 (1.01–1.04)^*^	1.04 (1.02–1.06)^*^
Residence	Rural	298	415	1	1
	Urban	86	26	4.61 (2.90–7.32)	3.18 (1.84–5.48)*
Educational level	Cannot read and write	138	202	1	1
	Can read and write	41	75	0.80 (0.52–1.24)	0.70 (0.43–1.14)
	Primary education	71	62	1.68 (1.12–2.51)^*^	2.02 (1.26–3.24)*
	Secondary education	59	77	1.12 (0.75–1.68)	1.12 (0.67–1.86)
	Diploma and above	75	25	4.39 (2.66–7.25)^*^	3.74 (1.99–7.02)*
Where salt was bought	Market	255	348	1	
	Nearby shop	129	93	1.89 (1.39–2.59)^*^	0.97 (0.66–1.44)
How salt was checked	I did not check	21	58	1	1
	By asking the seller	189	221	2.36 (1.38–4.04)^*^	2.21 (1.26–3.88)*
	By reading the pack	174	162	2.97 (1.72–5.11) ^*^	1.74 (0.93–3.24)
Is a cover used for the salt container?	No	94	155	1	1
	Yes	290	286	1.67 (1.23–2.27)^*^	1.58 (1.13–2.19)*
Place of salt storage	Damp area or close to a fire	14	37	1	1
	Dry and cool area	370	404	2.42 (1.29–4.55)^*^	2.72 (1.37–5.42)*
Duration of salt storage in household	More than 2 months	228	320	1	1
	Less than or equal to 2 months	136	121	1.45 (1.08–1.95)^*^	1.36 (0.99–1.88)
Knowledge about iodized salt and IDD	Poor	147	236	1	1
	Good	237	205	1.86 (1.41–2.45)^*^	1.22 (0.88–1.68)

NB: *Indicates (Significant at a *p*-value of <0.05), 1: reference group, COR: crude odds ratio, AOR: adjusted odds ratio, Hosmer and Lemeshow test = 0.11.

## Discussion

One of the most well-liked and effective public health initiatives for the global eradication of IDD is the universal iodization of salt (22). According to the legislation on salt released by the Ethiopian Council of Ministers in March 2011, all salt meant for human consumption must be iodized, and any such iodized salt must comply with the specifications for iodized salt stipulated by the appropriate authorities (23). However, this study found that 46.5% of households used adequate iodized salt (15 PPM), with a 95% confidence interval (CI) of [43–50.5%]. This research supports the findings of Jabitanan and West Gojjam (48.3%) (24).

The World Health Organization’s goal of eradicating iodine deficiency illnesses (≥90%) is substantially exceeded by this finding (1). According to the findings of this study, the proportion of households with adequate iodized salt is less than in earlier studies from Combolcha (68.8%) (25), Assella (62.5%) (26), and Dera (57.4%) (27), Oromia Regional State, South East Ethiopia (61.1%) (28), Southeast Ethiopia (56.6%) (28), Kolfe Keraniyo, Addis Ababa (95.5%) (29), Sidama Zone (65%) (30), China (89%) (31), Ghana (75.6%) (32), and India (75%) (33). This may be a result of the market’s accessibility and availability of iodized salt, as well as laws, policies, and ongoing follow-up and monitoring of the use of iodized salt in various nations.

The figures reported in this study, however, are higher than those that had previously been reported in Mecha (25.7%) (34), Dega Damot, West Gojjam (4.6%) (21), Jijiga town (40.8%) (14), Maychew, North Ethiopia (33%) (12), Gondar town (28.9%) (19), Addis Ababa (20%), Dire Dawa (7.5%) (35), Bale (32.7%) (36), Wollega (23.6%) (37), Arba Minch (41.8%) (38), Wolayita Sodo (37.7%) (20), and Volta region, Ghana (24.2%) (39). This variation may be due to a difference in the study’s execution period. The coverage of iodized salt increased from 28.4% in 2000 to 89% in 2016, according to the Ethiopian Demography and Health Survey (16). Even while national surveys show that the use of iodized salt in households has increased over time, the iodine content of the salt is still fairly low. Additionally, it could be the result of variations in the accessibility and availability of iodized salt on the market, iodine fortification regulations, and monitoring of iodized salt consumption in certain nations.

In this study, age, place of residence, level of education, checking of salt iodization while buying, place of salt storage, and cover use for salt containers were identified as significantly associated factors with the adequacy of iodized salt in the households. As age grew by one unit, the probability that the household had adequate iodized salt increased by 1.04 times. This result is supported by studies in Bahir Dar (40) and Arba Minch (38). This might be because age increases the ability to ask the seller, understand, and read the information on iodized salt.

The odds of having adequate iodized salt were 3.18 times higher in urban residents than in rural residents. This finding aligns with studies in Jibat Woreda, West Shoa Zone, Ethiopia (41), Nejo Woreda, Oromia Region, Ethiopia (42), and Bangladesh (43). This might be due to access to pure water, proper handling of salt, and checking of salt at the time of purchase. Urban residents had the opportunity to purchase and have a variety of sources that deliver the importance of iodized salt.

The odds of having adequately iodized salt in the household were 2.02 and 3.74 times higher in participants who attended primary education and had a diploma or higher, respectively. The result of studies done in Dera, Northwest Ethiopia (27), Mecha, Northwest Ethiopia (34), Jibat Woreda, West Shoa Zone, Ethiopia (41), Myichew Northern Ethiopia (12), Volta region Ghana (39), Bangladesh (43), and Pakistan (44) support this finding. This may be because education has the power to alter and read the levels of information on iodized salt. This and the fact that the majority of respondents mentioned a lack of sufficient knowledge and awareness of the benefits of iodized salt as their reasons for not purchasing it suggest that education facilitates access to and the use of iodized salt.

The study also found that the salt storage container and place were significant factors that affected how much iodized salt was adequate in the household. At the household level, edible salt can maintain more of its iodine content when stored in dry, covered containers. Other comparable studies carried out in the Lay Armachiho district and Gondar validated this conclusion (19, 45). This could be because salt that is kept in a moist environment collects moisture and becomes wet, which pushes the iodide component to the bottom of the container. Furthermore, if the container is opened when it’s hot, the salt may shed moisture from the surface, and as iodine is volatile, this could result in iodine loss.

Furthermore, households who asked the sellers about the iodization of the salt while buying salt had a 2.21 times higher likelihood of having adequate iodized salt than those who did not. This may be because members of the households do not understand or cannot comprehend the exact expiration date and iodized salt notifications. Similar research by Arba Minch (30) showed that the adequacy of iodized salt in household salt was not found to be associated with food handler knowledge or practice (30).

## Conclusion

The amount of iodized salt that is adequate for households in the district is less than what the WHO recommends. Additionally, we discovered that the existence of adequate iodized salt in a household was related to factors such as age, place of residence, level of education, checking of salt iodization while buying, place of salt storage, and cover use for salt containers. To increase the iodine content of salt at the household level, women should be encouraged to pursue education, and it should be recommended that households store their iodized salt in a dry environment in a closed container, and customers inquire about the seller or study the salt’s packaging when making their purchases.

### Strengths and limitations

This investigation used the gold standard iodometric titration measurement to determine the iodine content in households. However, this research was entirely quantitative. If it were combined with qualitative research, it might be more quantifiable. Therefore; future researchers will be conducting ca strong design and the most accurate and reliable urine concentration measurement. The use of urine concentration measurement increases the accuracy and dependability of sample measurement. Additional research also required to determine whether salt iodine loss occurs at the salt’s manufacturing facility, during transportation, or in the storage.

## Data availability statement

The original contributions presented in the study are included in the article/supplementary material, further inquiries can be directed to the corresponding author.

## Ethics statement

The studies involving humans were approved by Bahir Dar University, College of Medicine and Health Science. The studies were conducted in accordance with the local legislation and institutional requirements. The ethics committee/institutional review board waived the requirement of written informed consent for participation from the participants or the participants’ legal guardians/next of kin because Oral consent was obtained from each respondents. Written informed consent was obtained from the individual(s) for the publication of any potentially identifiable images or data included in this article.

## Author contributions

ET, OS, GE, YD, MB, TK, and AD made substantial contributions to conception and design, managing the data, or analysis and interpretation of data, took part in drafting the article or revising it critically for important intellectual content, agreed to submit it to the current journal, gave final approval of the version to be published, and agree to be accountable for all aspects of the work.

## Abbreviations

EDHS: Ethiopian Demographic and Health Survey
IDD: Iodine Deficiency Disorders
IDTM: Iodometric Titration Method
IRB: Institutional Review Board
NNP: National Nutrition Program
PPM: Parts Per Million
UNICEF: United Nations Children’s Fund
USI: Universal Salt Iodization
WHO: World Health Organization
